# Risk Factors and Reasons for Discharge Against Medical Advice for Newborns With Neonatal Surgical Diseases: A Preliminary Study From a Tertiary Care Hospital in Beijing, China

**DOI:** 10.3389/fped.2020.576270

**Published:** 2020-10-02

**Authors:** Shen Yang, Siyu Cai, Junmin Liao, Xiaoxia Peng, Jinshi Huang

**Affiliations:** ^1^Department of Neonatal Surgery, Beijing Children's Hospital, Capital Medical University, National Center for Children's Health, Beijing, China; ^2^Center for Clinical Epidemiology and Evidence-Based Medicine, Beijing Children's Hospital, Capital Medical University, National Center for Children's Health, Beijing, China

**Keywords:** discharge against medical advice (DAMA), newborns, neonatal surgical diseases, risk factors, reasons

## Abstract

**Background:** To identify the risk factors and reasons for discharge against medical advice (DAMA) for newborns with neonatal surgical diseases in a tertiary care hospital in China.

**Methods:** A retrospective study was conducted on all newborn patients admitted to the neonatal surgery department of Beijing Children's Hospital between January 1, 2016 and January 1, 2020. Medical records were compared between DAMA and non-DAMA patients. Univariate and multivariate logistic regression analyses were conducted to identify potentially useful characteristics for predicting DAMA.

**Results:** During the study period, 854 newborns were admitted to the neonatal surgery department. A total of 68 DAMA patients (68/854, 7.96%, 47 boys), with a median age at diagnosis of 1 day (range, from birth to 21 days), were included in this study. After multivariate analysis, we found that emergency admission, age at admission ≤5 days, rejection for surgery, and admission to the neonatal intensive care unit were significant independent risk factors for DAMA. According to the electronic medical records, the reasons for DAMA included belief in incurability and concerns about the prognosis of the disease (*n* = 31), multiple malformations with poor prognosis (*n* = 8), severe postoperative complications (*n* = 5), financial difficulties (*n* = 3), refusal of further examinations (*n* = 2), assumption of clinical improvement (*n* = 1), and unknown (*n* = 18).

**Conclusions:** This preliminary study showed that neonatal surgical patients in critical conditions were high-risk groups for DAMA, and the main possible reasons for DAMA were the parents' belief in incurability and concerns about the prognosis of the disease.

## Introduction

The neonatal period, a child's 1st month of life, is the most critical time for survival. Congenital malformations, preterm birth, neonatal sepsis, and injury remain leading causes of death (1). Congenital malformations, the most common neonatal surgical diseases, are a leading cause of mortality and morbidity in the neonatal period and infancy besides labor associated problems like asphyxia and prematurity (2). Approximately 2.5–5% of all neonates have at least one anatomical malformation (3), but the spectrum of this heterogeneous group of diseases is very wide.

In the department of neonatal surgery in developing countries, some parents of newborns with neonatal surgical diseases (especially congenital malformations) will choose to discharge against medical advice (DAMA) before or after the surgery. Possible reasons include the critical condition of the newborn, the combination of multiple malformations, family socio-economic factors, and concerns about the prognosis of the disease.

DAMA may lead to the worsening conditions or death of patients and increase the risk of readmission. Many studies focus on the abandonment of treatment and DAMA for children's cancer, and analyze the risk factors (4–6). However, few studies focus on DAMA for neonatal surgical diseases. This preliminary study aims to explore the relevant factors and reasons of DAMA by reviewing neonatal medical records from the department of neonatal surgery in our center.

## Methods

### Patients and Clinical Characteristics

We retrospectively collected the medical records of all newborn patients (28 days of age or younger) admitted to the neonatal surgery department of Beijing Children's Hospital between January 1, 2016 and January 1, 2020. Data on gender, age at admission, birth weight, gestational age, mode of delivery, father's, and mother's age at child's birth, gravidity and parity, geographic location, urgency of admission (patients with diseases such as congenital anorectal malformations, esophageal atresia, intestinal atresia, and abdominal wall deformity, as well as patients with gastrointestinal perforation, intestinal obstruction, bleeding, dehydration, acute infection and other complications, were usually admitted from the emergency department), admission condition, diagnosis, treatment of surgery and operative time, re-operation during the first hospitalization, admission to the neonatal intensive care unit (indications included premature and low birth weight infants, patients requiring mechanical ventilation, patients after cardiopulmonary resuscitation, and so on), length of stay, hospitalization expense, and readmission were collected. Other information was also collected for DAMA patients, including the condition of siblings, whether multiple malformations, congenital heart disease, chromosome abnormality, or VACTERL syndrome were present, and the reasons for DAMA. All methods were carried out in accordance with relevant guidelines and regulations, and the study was approved by the Medical Ethics Committee of Beijing Children's Hospital (2020-Z-039). A waiver of consent was provided for the analyses conducted in this study.

### Statistical Analysis

Statistical analysis was performed by SAS 9.4. Continuous variables were presented as the mean and standard deviation for normal distribution, or median and interquartile range for non-normal distribution. Categorical variables were reported as counts and percentages. Two independent samples *t*-tests and χ^2^ tests were used to compare characteristics between the DAMA and non-DAMA groups. Receiver operating characteristic (ROC) curve analysis was performed to determine the most appropriate cut-off values. Univariate and multivariate logistic regression analyses were conducted to select potentially useful characteristics for predicting DAMA. *P* < 0.05 was considered statistically significant.

## Results

### Comparison Between DAMA and Non-DAMA Groups

During the study period from January 2016 to December 2019, 854 newborn patients (28 days of age or younger) were admitted to the neonatal surgery department of Beijing Children's Hospital. A total of 68 DAMA patients (68/854, 7.96%, 47 boys and 21 girls), with a median age at diagnosis of 1 day (range, from birth to 21 days), were included in this study. As shown in Table 1, by comparing medical records between DAMA (*n* = 68) and non-DAMA (*n* = 786) patients, we found significant differences in age at admission, birth weight, urgency of admission, admission condition, treatment of surgery, admission of neonatal intensive care unit, length of stay, and hospitalization expense (all *P* < 0.05). However, there were no differences in gender, gestational age, mode of delivery, father's, and mother's age at child's birth, parity and gravidity, geographic location, re-operation during the first hospitalization, operative time, and readmission between the two groups (all *P* > 0.05). The disease distribution of the two groups is shown in Table 2.

**Table 1:** Comparison between DAMA and non-DAMA groups.

**Variables**		**DAMA (*n* = 68)**	**Non-DAMA (*n* = 786)**	**Results**	***p***
Gender (*n*, %)	Boy	47 (69.12)	499 (63.49)	0.8610	0.3535
	Girl	21 (30.88)	287 (36.51)		
Age at admission (median, days)		1.00 (1.00, 3.00)	3.00 (1.00, 15.00)	−4.6987	<0.0001
Birth weight (median, g)		2,955.00 (2,562.50, 3,400.00)	3,240.00 (2,900.00, 3,590.00)	−3.2593	0.0011
Gestational age (*n*, %)	Preterm	9 (13.24)	85 (12.28)	0.052	0.8200
	Term	59 (86.76)	607 (87.72)		
Mode of delivery (*n*, %)	Cesarean delivery	38 (55.88)	336 (48.55)	1.3300	0.2488
	Spontaneous vaginal delivery	30 (44.12)	356 (51.45)		
Father's age at child's birth (median, years)		30 (27, 34)	31 (28, 34)	−1.5955	0.1106
Mother's age at child's birth (median, years)		29 (27, 32)	30 (27, 33)	−1.0713	0.2840
Gravidity (median)		2 (1,3)	2 (1,2)	0.8923	0.3722
Parity (median)		1 (1,2)	2 (1,2)	−1.3663	0.1719
Geographic location (*n*, %)	City	40 (58.82)	423 (53.82)	0.6320	0.4266
	Village	28 (41.18)	363 (46.18)		
Urgency of admission (*n*, %)	Emergency	55 (80.88)	442 (56.23)	15.6290	<0.0001
	Planned	13 (19.12)	344 (43.77)		
Admission condition (*n*, %)	Critical	50 (74.63)	405 (51.59)	13.1630	0.0003
	Stable	17 (25.37)	380 (48.41)		
Surgery (*n*, %)	No	25 (36.76)	59 (7.51)	60.4110	<0.0001
	Yes	43 (63.24)	727 (92.49)		
Re-operation during the first hospitalization (*n*, %)	No	67 (98.53)	769 (97.84)	0.1450	0.7030
	Yes	1 (1.47)	17 (2.16)		
Operative time (median, min)		75.00 (52.75, 117.25)	68.00 (48.00, 95.00)	1.4996	0.1337
Neonatal intensive care unit (*n*, %)	No	52 (76.47)	704 (89.57)	10.5680	0.0012
	Yes	16 (23.53)	82 (10.43)		
Length of stay (median, days)		3.50 (1.00, 14.00)	13.00 (8.00, 19.00)	−5.9985	<0.0001
Hospitalization expense (median, CNY)		12654.03 (3857.53, 33802.47)	21840.18 (15272.80, 29655.49)	−3.9819	<0.0001
Readmission	No	59 (86.76)	665 (84.61)	0.2260	0.6344
	Yes	9 (13.24)	121 (15.39)		

**Table 2:** The disease distribution of the DAMA and non-DAMA groups.

**Discharge diagnosis**	**DAMA (*****n*** **=** **68)**		**Non-DAMA (*****n*** **=** **786)**	
	**Yes (*n*, %)**	**No (*n*, %)**	**Yes (*n*, %)**	**No (*n*, %)**
Congenital anorectal malformation	17 (25.00)	51 (75.00)	166 (21.12)	620 (78.88)
Esophageal atresia/tracheoesophageal fistula	13 (19.12)	55 (80.88)	34 (4.33)	752 (95.67)
Congenital intestinal atresia	11 (16.18)	57 (83.82)	66 (8.40)	720 (91.60)
Hirschsprung's disease/Hirschsprung's allied disease	10 (14.71)	58 (85.29)	105 (13.36)	681 (86.64)
Congenital duodenal obstruction	3 (4.41)	65 (95.59)	75 (9.54)	711 (90.46)
Congenital malrotation of the intestine	3 (4.41)	65 (95.59)	58 (7.38)	728 (92.62)
Tumor	2 (2.94)	66 (97.06)	80 (10.18)	706 (89.82)
Intestinal obstruction, perforation of digestive tract, peritonitis	2 (2.94)	66 (97.06)	50 (6.36)	736 (93.64)
Congenital abdominal wall malformation	2 (2.94)	66 (97.06)	36 (4.58)	750 (95.42)
Encephalocele and meningocele	2 (2.94)	66 (97.06)	5 (0.64)	781 (99.36)
Congenital hypertrophic pyloric stenosis	0 (0)	68 (100.00)	50 (6.36)	736 (93.64)
Congenital bile duct malformation	0 (0)	68 (100.00)	8 (1.02)	778 (98.98)
Necrotic enterocolitis of newborn	0 (0)	68 (100.00)	5 (0.64)	781 (99.36)
Congenital diaphragmatic hernia	0 (0)	68 (100.00)	4 (0.51)	782 (99.49)
Others	3 (4.41)	65 (95.59)	44 (5.60)	742 (94.40)

### Risk Factors for DAMA

In order to find the risk factors for DAMA, we further conducted a multivariate analysis. ROC curve analysis was used to determine the stratification value for age at admission and birth weight according to the maximum combined sensitivity and specificity values. The cut-off values for the above characteristics were 5 days and 3,000 g, respectively (Supplementary Figure 1). As shown in Table 3, multivariate analysis showed that emergency admission, age at admission ≤5 days, rejection for surgery, and admission to the neonatal intensive care unit were significant independent risk factors for DAMA.

**Table 3:** Multivariate logistic regression analysis of prediction of DAMA.

**Variables**	**Estimate**	**Standard Error**	**Wald**	***P***	**OR**
Emergency admission	0.7943	0.3394	5.4791	0.0192	2.213 (1.138, 4.304)
Age at admission ≤5 days	1.3284	0.3862	11.8335	0.0006	3.775 (1.771, 8.047)
Rejection for surgery	2.3946	0.3249	54.3264	<0.0001	0.091 (0.048, 0.172)
Admission to the neonatal intensive care unit	1.1271	0.3431	10.7932	0.0010	3.087 (1.576, 6.046)

### Clinical Characteristics of DAMA Patients

As shown in Table 4, among the 68 DAMA neonates, 4 were twins, 28 had an older sibling (one older sibling had died of severe congenital malformation after birth and another died of intracerebral hemorrhage after birth), 30 had multiple malformations, 13 had congenital heart disease, 5 had chromosome abnormalities, and 5 had VACTERL syndrome. According to the electronic medical records, the reasons for 68 DAMA newborns included belief in incurability and concerns about the prognosis of the diseases (*n* = 31), multiple malformations with poor prognosis (*n* = 8), severe postoperative complications (*n* = 5), financial difficulties (*n* = 3), refusal of further examinations (*n* = 2), assumption of clinical improvement (*n* = 1), and unknown (*n* = 18).

**Table 4:** The clinical characteristics of the DAMA patients.

**Variables**		**Number (*****n*** **=** **68)**	
		**Yes (*n*, %)**	**No (*n*, %)**
Twin		4 (5.88)	64 (94.12)
Elder brother or sister		28 ^*^ (41.18)	40 (58.82)
Multiple malformations		30 (44.12)	38 (55.88)
Congenital heart disease		13 (19.12)	55 (80.88)
Chromosome abnormality		5 (7.35)	63 (92.65)
VACTERL syndrome		5 (7.35)	63 (92.65)
Reasons for DAMA	Belief in incurability and concerns about the prognosis of the diseases	31 (45.59)	37 (54.41)
	Multiple malformations with poor prognosis	8 (11.76)	60 (88.24)
	Severe postoperative complications	5 (7.35)	63 (92.65)
	Financial difficulties	3 (4.41)	65 (95.59)
	Refusal of further examinations	2 (2.94)	66 (97.06)
	Assumption of clinical improvement	1 (1.47)	67 (98.53)
	Unknown	18 (26.47)	50 (73.53)

* *One older sibling had died of severe congenital malformation after birth and another died of intracerebral hemorrhage after birth*.

## Discussion

As far as we know, this is the first study to explore the risk factors and reasons for DAMA newborns with neonatal surgical diseases. This preliminary study showed that neonatal surgical patients in critical conditions (emergency admission, age at admission ≤5 days, rejection for surgery, and admission to the neonatal intensive care unit) were high-risk groups for DAMA, and the main possible reasons for DAMA were the parents' belief in incurability and concerns about their respective prognosis, and multiple malformations with poor prognosis.

DAMA is a matter of grave concern and a challenge routinely faced by hospital treatment teams. Patients who leave medical facilities before their treatment is completed and against doctors' approval tend to deteriorate. However, these incidents are difficult to eliminate and is prevalent in almost all healthcare facilities, leading to unpredictable complications (7). DAMA rates vary across countries, age groups, diseases, and hospital departments. Among neonates, previous studies have reported that the proportion of DAMA is between 1.6 and 25.4% (8–10). First week of life, low socioeconomic class and education of parents have been reported as risk factors of newborns who subsequently DAMA (9). Common reasons identified for DAMA among children in former studies included parents' false perception that their child's condition had improved; difficulties arranging care for the patient's siblings at home; perception of poor clinical outcome; logistical reasons such as living far from the hospital, or parents living outside the province; unavailability of consultation appointment; dissatisfaction with treatment; discomfort caused by frequent blood tests for laboratory investigations; child's refusal to stay at the hospital; prolonged hospitalization period causing financial problems, inconvenience (lost work days), and preference for traditional healers (7–9). However, in our study, the main reasons for DAMA were belief in incurability and concerns about the prognosis of the diseases, and multiple malformations with poor prognosis.

Parents of pediatric patients are faced with a different challenge when opting for DAMA, since patients of this particular age group have neither the required emotional and cognitive maturity nor the legal rights to decide for themselves. Former studies showed that the leading diagnoses in cases of neonatal DAMA were sepsis, asphyxia, prematurity, and jaundice (8, 9). These diagnoses have also been identified by WHO as the greatest cause of mortality of newborns in the developing world. However, the top three causes of DAMA in this study were congenital anorectal malformations, esophageal atresia/tracheoesophageal fistula, and congenital intestinal atresia. On the one hand, these diseases are usually accompanied by a variety of other malformations, such as congenital heart disease, urogenital system malformation, spinal deformity, etc., and patients often need to undergo multiple operations. On the other hand, these diseases are associated with a relatively high incidence of short-term and long-term complications after surgery which can also seriously affect the quality of life. Examples include fecal and urinary incontinence and sexual inadequacy after repair of high-type anorectal malformation (11), anastomotic stricture, long-term gastrointestinal, and respiratory complications and comorbidities after repair of long gap esophageal atresia (12), and short bowel syndrome after repair multiple intestinal atresia (13). When parents comprehend the course of treatment and the long-term prognosis of the diseases, many parents state that they cannot tolerate the repeated surgical procedures and the uncertain therapeutic effects, especially when the newborn is burdened with multiple malformations.

Socio-economic status of parents is considered one of the major factors related to DAMA in most of the studies conducted on self-discharged neonates. Former studies showed that 54.8–72.1% parents who self-discharged their child had a lower socio-economic status (9). However, our study was unable to obtain the relevant socio-economic data of the patients from the medical records. Although no statistical difference was found between the geographic location of city or village between the DAMA and non-DAMA groups, many parents did choose to self-discharge their babies due to financial difficulties in our study. In order to solve the problem of children who come from financially unstable families being unable to get medical treatment, China has been vigorously implementing medical insurance policies in urban and rural areas for many years. In addition, China implemented a program to assist pediatric patients with congenital structural abnormalities in 2017, and by 2020, more than 15,000 patients nationwide have benefited.

Previous studies have shown that the readmission rate of DAMA patients is significantly higher than non-DAMA patients (8). This phenomenon was not found in our study and a possible reason could be that most parents of DAMA newborns chose to refuse treatment. The Hippocratic oath binds a physician to uphold the patients' well-being. However, pediatricians can sometimes be conflicted between deciding on the best clinical course for the child and the parents' decision to discharge their child. Each healthcare professional can play a vital role in preventing DAMA. A good starting point would be the administration of proper DAMA case documentation whereby the underlying reason for DAMA be clearly recorded. This documentation may then be used for legal purposes, and should also be used for potential interventions aimed at reducing DAMA. Additionally, more effective communication and counseling are required between parents and the pediatricians. This may help avoid DAMA and prevent potential damages to the children's health.

Despite the enormous clinical, economic, ethical, and medico-legal implications of DAMA especially in developing countries, it has largely remained an under researched area, particularly in neonates. Limitation of the present study was inability to follow up on DAMA babies to determine their outcome. Detailed DAMA information could not be obtained because a DAMA registry has not yet been established in our hospital. Moreover, the reasons of DAMA may vary according to specific diseases. Within a particular disease, differing development of economy and medical levels will naturally affect the parents' level of understanding of the disease, thereby also affecting the reasons for DAMA. Therefore, based on this preliminary study, we plan to facilitate qualitative research in the future to gain an in-depth understanding of the causes of DAMA newborns with neonatal surgical diseases, and further formulate corresponding policies to minimize the occurrence of DAMA.

In conclusion, this study provides us with a better understanding of the risk factors and reasons for DAMA newborns with neonatal surgical diseases. Understanding these factors can encourage appropriate interventions and policies for reducing DAMA rates in the future. In this way, pediatric patients can be protected from the unfortunate consequences of inappropriate discharge.

## Data Availability Statement

The original contributions presented in the study are included in the article/Supplementary Material, further inquiries can be directed to the corresponding author/s.

## Ethics Statement

The studies involving human participants were reviewed and approved by Medical Ethics Committee of Beijing Children's Hospital (2020-Z-039). Written informed consent from the participants' legal guardian/next of kin was not required to participate in this study in accordance with the national legislation and the institutional requirements.

## Author Contributions

Study conception and design: JH and SY. Data acquisition: JL. Analysis and data interpretation: SC and XP. Drafting of the manuscript: SY. Contributed to the article and approved the submitted version: All authors.

## Conflict of Interest

The authors declare that the research was conducted in the absence of any commercial or financial relationships that could be construed as a potential conflict of interest.

## Supplementary Material

Supplementary Figure 1 ROC curve analyses. Stratification values for **(A)** age at admission and **(B)** birth weight, which were calculated by ROC curve analyses.
