# Supplementary figures and images for: Risk Factors and Reasons for Discharge Against Medical Advice for Newborns With Neonatal Surgical Diseases: A Preliminary Study From a Tertiary Care Hospital in Beijing, China
Source: Front Pediatr. 2020 Oct 2;8:576270. doi: 10.3389/fped.2020.576270 (PMC7562829; doi:10.3389/fped.2020.576270)

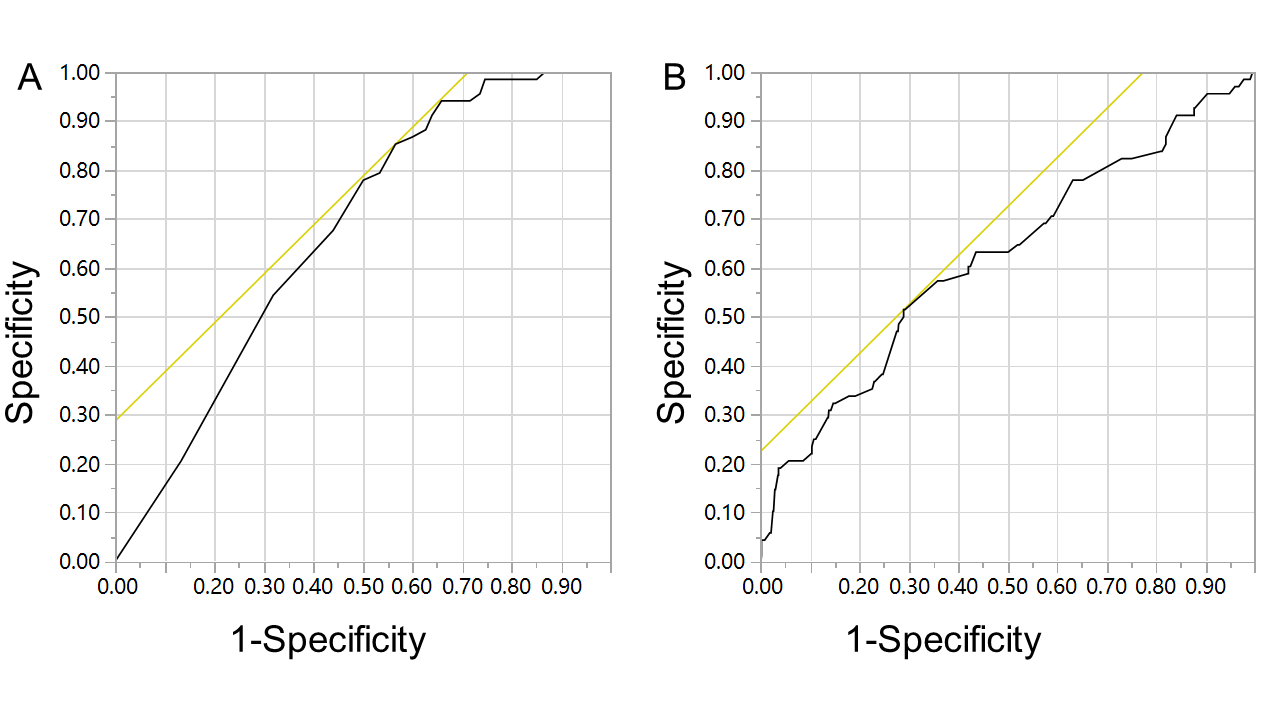

Supplement: Supplementary Figure 1 — ROC curve analyses. Stratification values for (A) age at admission and (B) birth weight, which were calculated by ROC curve analyses. [file Image_1.TIF]
